# Supplementary material for: HIVprotI: an integrated web based platform for prediction and design of HIV proteins inhibitors
Source: J Cheminform. 2018 Mar 9;10:12. doi: 10.1186/s13321-018-0266-y (PMC5845081; doi:10.1186/s13321-018-0266-y)
Supplement: Supplementary file 2 — Additional file 2. Source code of HIVProtI web server. [file 13321_2018_266_MOESM2_ESM.zip › HIVprotI_Source-code/hivprotI/weka-3-6-6/doc/weka/classifiers/meta/RandomCommittee.html]

RandomCommittee


---


|  |  |  |
| --- | --- | --- |
| **Overview** \| **Package** \| **Class** \| **Tree** \| **Deprecated** \| **Index** \| **Help** \| **Weka's home** | |  |
| **PREV CLASS**   **NEXT CLASS** | **FRAMES**    **NO FRAMES**     **All Classes** |  |
| SUMMARY: NESTED \| FIELD \| CONSTR \| METHOD | DETAIL: FIELD \| CONSTR \| METHOD |  |


---


## weka.classifiers.meta Class RandomCommittee

```
java.lang.Object
  weka.classifiers.Classifier
      weka.classifiers.SingleClassifierEnhancer
          weka.classifiers.IteratedSingleClassifierEnhancer
              weka.classifiers.RandomizableIteratedSingleClassifierEnhancer
                  weka.classifiers.meta.RandomCommittee
```

**All Implemented Interfaces:**: java.io.Serializable, java.lang.Cloneable, CapabilitiesHandler, OptionHandler, Randomizable, RevisionHandler, WeightedInstancesHandler

---

``` public class RandomCommittee extends RandomizableIteratedSingleClassifierEnhancer implements WeightedInstancesHandler ```

Class for building an ensemble of randomizable base classifiers. Each base classifiers is built using a different random number seed (but based one the same data). The final prediction is a straight average of the predictions generated by the individual base classifiers.


Valid options are:

```
 -S <num>
  Random number seed.
  (default 1)
```

```
 -I <num>
  Number of iterations.
  (default 10)
```

```
 -D
  If set, classifier is run in debug mode and
  may output additional info to the console
```

```
 -W
  Full name of base classifier.
  (default: weka.classifiers.trees.RandomTree)
```

```
 Options specific to classifier weka.classifiers.trees.RandomTree:
```

```
 -K <number of attributes>
  Number of attributes to randomly investigate
  (<1 = int(log(#attributes)+1)).
```

```
 -M <minimum number of instances>
  Set minimum number of instances per leaf.
```

```
 -S <num>
  Seed for random number generator.
  (default 1)
```

```
 -depth <num>
  The maximum depth of the tree, 0 for unlimited.
  (default 0)
```

```
 -D
  If set, classifier is run in debug mode and
  may output additional info to the console
```

Options after -- are passed to the designated classifier.

**Version:**
:   $Revision: 1.13 $

**Author:**
:   Eibe Frank (eibe@cs.waikato.ac.nz)

**See Also:**: Serialized Form

---

**Constructor Summary**

`RandomCommittee()`
  
          Constructor.


| **Method Summary** | |
| --- | --- |
| `void` | `buildClassifier(Instances data)`             Builds the committee of randomizable classifiers. |
| `double[]` | `distributionForInstance(Instance instance)`             Calculates the class membership probabilities for the given test instance. |
| `java.lang.String` | `getRevision()`             Returns the revision string. |
| `java.lang.String` | `globalInfo()`             Returns a string describing classifier |
| `static void` | `main(java.lang.String[] argv)`             Main method for testing this class. |
| `java.lang.String` | `toString()`             Returns description of the committee. |

**Methods inherited from class weka.classifiers.RandomizableIteratedSingleClassifierEnhancer**

`getOptions, getSeed, listOptions, seedTipText, setOptions, setSeed`

**Methods inherited from class weka.classifiers.IteratedSingleClassifierEnhancer**

`getNumIterations, numIterationsTipText, setNumIterations`

**Methods inherited from class weka.classifiers.SingleClassifierEnhancer**

`classifierTipText, getCapabilities, getClassifier, setClassifier`

**Methods inherited from class weka.classifiers.Classifier**

`classifyInstance, debugTipText, forName, getDebug, makeCopies, makeCopy, setDebug`

**Methods inherited from class java.lang.Object**

`equals, getClass, hashCode, notify, notifyAll, wait, wait, wait`

**Constructor Detail**

### RandomCommittee

```
public RandomCommittee()
```

:   Constructor.


**Method Detail**

### globalInfo

```
public java.lang.String globalInfo()
```

:   Returns a string describing classifier

    :   **Returns:**: a description suitable for displaying in the explorer/experimenter gui

---


### buildClassifier

```
public void buildClassifier(Instances data)
                     throws java.lang.Exception
```

:   Builds the committee of randomizable classifiers.

    :   **Overrides:**: `buildClassifier` in class `IteratedSingleClassifierEnhancer`
    :   **Parameters:**: `data` - the training data to be used for generating the bagged classifier. **Throws:**: `java.lang.Exception` - if the classifier could not be built successfully

---


### distributionForInstance

```
public double[] distributionForInstance(Instance instance)
                                 throws java.lang.Exception
```

:   Calculates the class membership probabilities for the given test
    instance.

    :   **Overrides:**: `distributionForInstance` in class `Classifier`
    :   **Parameters:**: `instance` - the instance to be classified **Returns:**: preedicted class probability distribution **Throws:**: `java.lang.Exception` - if distribution can't be computed successfully

---


### toString

```
public java.lang.String toString()
```

:   Returns description of the committee.

    :   **Overrides:**: `toString` in class `java.lang.Object`
    :   **Returns:**: description of the committee as a string

---


### getRevision

```
public java.lang.String getRevision()
```

:   Returns the revision string.

    :   **Specified by:**: `getRevision` in interface `RevisionHandler` **Overrides:**: `getRevision` in class `Classifier`
    :   **Returns:**: the revision

---


### main

```
public static void main(java.lang.String[] argv)
```

:   Main method for testing this class.

    :   **Parameters:**: `argv` - the options


---


|  |  |  |
| --- | --- | --- |
| **Overview** \| **Package** \| **Class** \| **Tree** \| **Deprecated** \| **Index** \| **Help** \| **Weka's home** | |  |
| **PREV CLASS**   **NEXT CLASS** | **FRAMES**    **NO FRAMES**     **All Classes** |  |
| SUMMARY: NESTED \| FIELD \| CONSTR \| METHOD | DETAIL: FIELD \| CONSTR \| METHOD |  |


---
